# Using tiered accreditation and decentralized governance to achieve population-based equity in pulmonary and critical care medicine in the Chinese mainland

**DOI:** 10.1038/s43856-026-01724-1

**Published:** 2026-06-10

**Authors:** Naijian Li, Xiaolin Ye, Qiongdan Zhang, Xiaojing Liu, Huanqing Lai, Yu Pan, Shiyue Li, Weixin Chen

**Affiliations:** 1 Department of Allergy and Clinical Immunology, State Key Laboratory of Respiratory Disease, National Center for Respiratory Medicine, Guangzhou Institute of Respiratory Health, The First Affiliated Hospital of Guangzhou Medical University, Guangzhou, PR China; 2 Guangzhou Medical University, Guangzhou, PR China; 3 Pulmonary and Critical Care Medicine, State Key Laboratory of Respiratory Disease, National Center for Respiratory Medicine, Guangzhou Institute of Respiratory Health, The First Affiliated Hospital of Guangzhou Medical University, Guangzhou, PR China; 4 Faculty of Chinese Medicine, Macau University of Science and Technology, Macao, PR China

**Keywords:** Population screening, Nutritional supplements

## Abstract

**Background:**

Pulmonary and critical care medicine has become essential for managing severe respiratory conditions, yet many countries struggle to expand such services equitably across regions. Since 2018, the Chinese mainland has pursued a national strategy to establish specialized pulmonary and critical care departments using flexible accreditation standards that allow hospitals to progress at differently based on local capacity. The extent to which this approach has achieved equitable distribution remains unknown.

**Methods:**

We analyzed data from 31 provincial-level regions in the Chinese mainland from 2018 to 2023, from the national pulmonary and critical care registry, health statistical yearbooks, and census databases. We assessed distribution equity using Lorenz curves, Gini coefficients, and a newly developed density index that accounts for both population and geographic area. Geographical Detector analysis was used to assess socioeconomic and institutional factors associated with spatial disparities.

**Results:**

Here we show that the number of pulmonary and critical care departments increases by 470.9% (from 409 to 2335), with the fastest growth occurring in underserved regions. Population-based equity improves (Gini coefficient decreasing from 0.288 to 0.169). However, geographic disparities remain severe (Gini: 0.644), particularly in western provinces. Factors showing the strongest spatial correspondence with these disparities include government health expenditure, public hospital availability, and interactions between economic development and administrative capacity. Workforce ratios (nurse-to-physician: 1.96) remain below international benchmarks.

**Conclusions:**

The tiered accreditation model implemented in the Chinese mainland demonstrates that flexible, decentralized implementation can achieve rapid and population-equitable expansion of specialized medical services. These findings offer transferable principles for other countries seeking to balance national standardization with local adaptability, though addressing persistent geographic disparities and workforce shortages requires attention.

## Abstract (web summary)

Li *et al*. analyze nationwide data from 31 Chinese provinces (2018–2023) from the national pulmonary and critical care registry, health statistical yearbooks, and census databases. They find increasing pulmonary and critical care departments and improving population-based equity in access but geographical disparities remain.

## Plain Language Summary

The Chinese mainland has rapidly expanded specialized respiratory and critical care (PCCM) departments since 2018, but it remains unclear whether this growth benefited all regions equally. We analyzed national data to assess how the Chinese mainland’s approach, which combines flexible accreditation standards with local decision-making, affects the distribution of these departments across the country. We find that the number of PCCM departments increased nearly fivefold, from 409 to 2,335, and their distribution became more equitable if population size is considered. However, geographic disparities persist with remote and sparsely populated areas still lacking access. These findings demonstrate that flexible policies can achieve rapid healthcare expansion without compromising population-level equity. For the public, this means that well-designed national strategies can help ensure that even underserved communities gain access to specialized medical care, though challenges remain in reaching the most geographically isolated populations.

## Introduction

The transition from traditional respiratory medicine to pulmonary and critical care medicine (PCCM) represents a significant advancement in integrating respiratory care with critical care^1^. This evolution not only improves the management of respiratory disorders but also enhances the ability to address severe and life-threatening conditions, reflecting the growing demand for multidisciplinary and intensive care expertise. Globally, PCCM has become an essential component of healthcare systems, particularly in regions with a high burden of respiratory diseases and associated mortality. The adoption of the PCCM model in the Chinese mainland, inspired by the established U.S. framework, represents a strategic response to these challenges and opportunities^2–4^.

In 2008, the Chinese Medical Doctor Association initiated efforts to replicate the U.S. PCCM model, recognizing its potential to enhance patient outcomes and streamline respiratory and critical care delivery^5^. This initiative aligned with broader healthcare reform efforts in the Chinese mainland, emphasizing specialization, quality improvement, and equitable access to medical services. Over the next decade, these efforts culminated in the publication of the Standards for the Standardized Construction of PCCM departments in Secondary and Tertiary Hospitals in 2018^6,7^. This milestone was the result of collaboration among key organizations, including the Chinese Association of Chest Physicians (CACP), the Chinese Thoracic Society, the National Respiratory Specialist Consortium, and the National Center for Respiratory Medicine Quality Control, establishing a unified framework for PCCM development across the Chinese mainland.

PCCM development in the Chinese mainland has been both incremental and transformative, reflecting its unique healthcare landscape and evolving priorities^8^. Significant progress has been made in workforce development, particularly through specialist training programs. However, challenges persist, including regional disparities, resource allocation, and the optimization of department infrastructure to address the needs of diverse populations. Despite these advances, research on PCCM development in the Chinese mainland remains limited, particularly regarding the broader socioeconomic, institutional, and policy-related factors influencing its growth^9–11^.

In this study, we analyze data from 31 provincial-level regions in the Chinese mainland (2018–2023) to evaluate the distribution and determinants of PCCM department expansion. Here we show that the number of PCCM departments increases nearly fivefold, with the fastest growth occurring in historically underserved regions. Population-based equity substantially improves, although geographic disparities persist. These findings demonstrate that flexible accreditation systems can achieve rapid and equitable expansion of specialized medical services, offering transferable principles for other countries.

## Methods

### Data source

Data on PCCM departments in the Chinese mainland from 2018 to 2023 were obtained from three primary sources: the official China PCCM website, the China Health Statistics Yearbook published by the National Health Commission, and the China Statistical Yearbook published by the National Bureau of Statistics^12–14^. Administrative boundary data for map generation were obtained from the National Geographic Information Public Service Platform and released by the National Geomatics Center of China under map review approval no. GS(2024)0650. Information on physicians, nurses, and bed counts is obtained from respective hospital websites, with data prior to 2018 being excluded (Table 3 in Supplementary Data 2 in the Online Data Supplement). Under the PCCM accreditation standards in mainland China, essential facilities were required for certification; therefore, facility integration was not analyzed as a separate variable in this study. The distribution of PCCM departments is shaped by a range of factors, including demographic, economic, medical, educational, and administrative variables. Demographic factors encompass resident population size (X_1_), population density (X_2_), and the proportion of the urban population (X_3_). Economic factors include GDP (X_4_) and per capita disposable income (X_5_). Hospital-related factors comprise the number of tertiary hospitals (X_6_) and public hospitals (X_7_). Medical insurance factors cover insurance density (X_8_) and depth (X_9_). Medical expenditure factors include household healthcare spending (X_10_), government health expenditure (X_11_), and total health costs (X_12_). Educational factors consider the proportion of the population with a college degree or higher (X_13_) and the number of medical colleges (X_14_). Lastly, administrative division factors are represented by the number of prefecture-level cities (X_15_) (Supplementary Data 1). These province-level indicators were included as candidate stratifying factors in the geographical detector analysis to assess spatial correspondence with provincial variation in PCCM department distribution.

### Ethics approval

Not applicable. This study is a secondary analysis of publicly available data and does not involve human participants, human biological material, or identifiable private information. All data were obtained from publicly accessible sources, including the China PCCM registry, the China Health Statistics Yearbook, the China Statistical Yearbook, and publicly accessible hospital websites. No individual-level health records, human biological material, or identifiable private information were used. These datasets are freely accessible without registration or application requirements. No additional IRB approval was sought or obtained, consistent with institutional policies for research using publicly available aggregated data.

### Setting

**Fig. 1:**
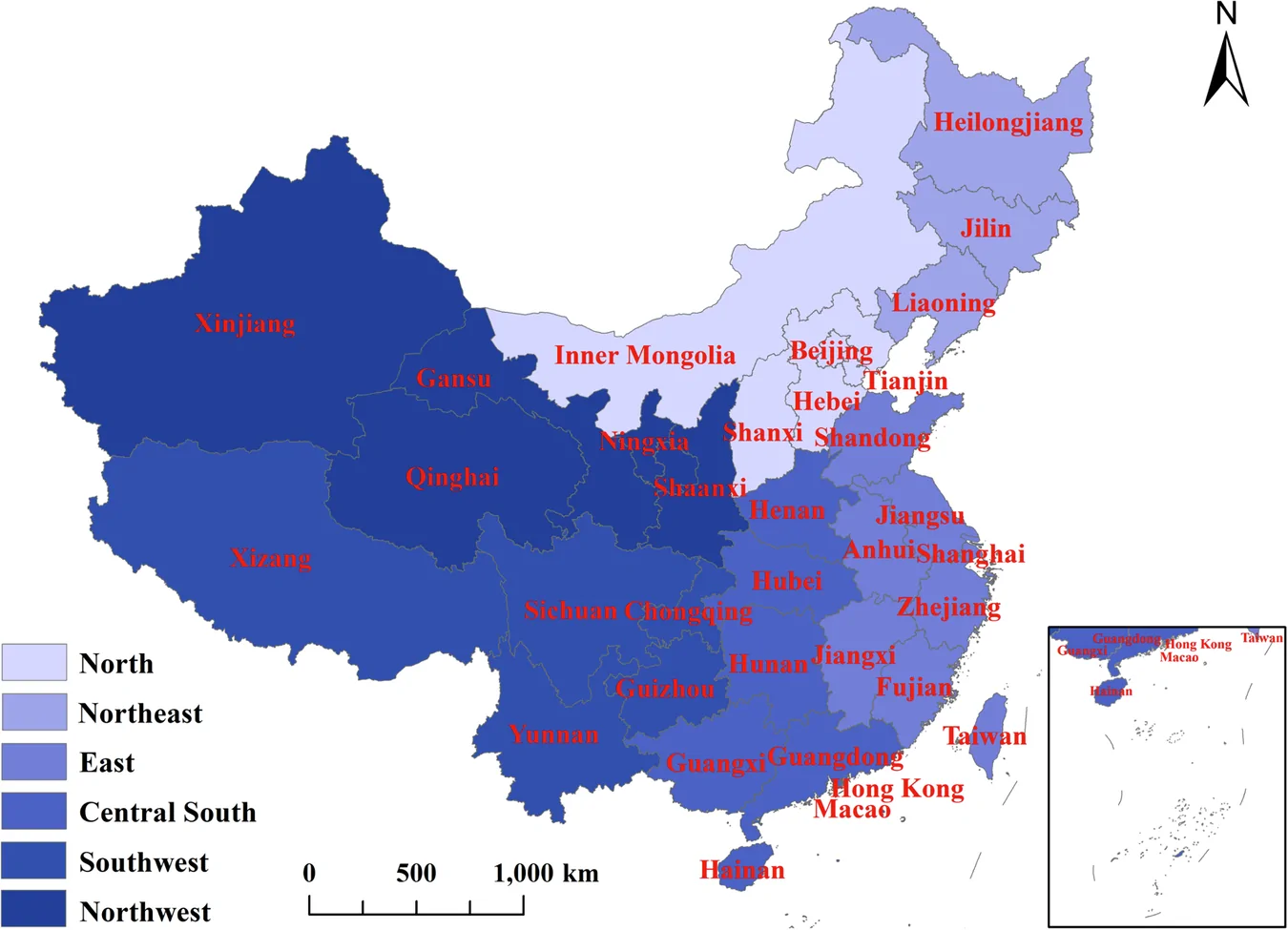
Geographic distribution of six administrative regions in the Chinese mainland. This figure illustrates the division of the Chinese mainland into six major administrative regions-North, Northeast, East, Central South, Southwest, and Northwest-based on historical, geographical, economic, and ethnic factors, providing a framework for analyzing regional disparities in PCCM distribution. The base geographic boundary data for this map were obtained from the National Geographic Information Public Service Platform and released by the National Geomatics Center of China under map review approval no. GS(2024)0650.

This study included 31 provincial-level administrative regions in the Chinese mainland. Hong Kong, Macao, and Taiwan were excluded because comparable data were unavailable. These 31 provincial-level regions are typically categorized into six major regions based on historical, geographical, economic, and ethnic factors: North (Beijing, Tianjin, Hebei, Shanxi, and Inner Mongolia), Northeast (Liaoning, Jilin, and Heilongjiang), East (Shanghai, Jiangsu, Zhejiang, Anhui, Fujian, Jiangxi, and Shandong), Central South (Henan, Hubei, Hunan, Guangdong, Guangxi, and Hainan), Southwest (Chongqing, Sichuan, Guizhou, Yunnan, and Xizang), and Northwest (Shaanxi, Gansu, Qinghai, Ningxia, and Xinjiang) (Fig. 1).

### Data analysis

Descriptive statistical methods were used to summarize the changes in the distribution of PCCM departments in the Chinese mainland from 2018 to 2023 and to assess the development status of PCCM departments in 2023. The distribution map of PCCM departments in the Chinese mainland from 2018 to 2023 was created using ArcGIS Pro 3.1. Lorenz curves, Gini coefficients, Theil indices, and the PCCM Density Index (PCCMDI), based on population and geographic areas, were employed to evaluate the inequity of PCCM departments' distribution in the Chinese mainland. These three indices were selected for their complementary strengths: the Gini coefficient captures overall distribution inequality, the Theil index enables decomposition of inter-regional and intra-regional disparities, and the PCCMDI integrates population and geographic area to reflect resource accessibility, ensuring a comprehensive multi-dimensional equity assessment. Geodetector analysis was conducted to explore the factors associated with spatial heterogeneity in PCCM departments across the Chinese mainland.

### The Lorenz curve

The Lorenz curve, introduced by American statistician Max O. Lorenz in 1905, was originally developed to illustrate income inequality^15^. Over time, its application has expanded to diverse fields, including the evaluation of healthcare resource distribution^16^. In this context, the Lorenz curve offers a robust framework for assessing the equity in the distribution of PCCM departments in the Chinese mainland. The area between the Lorenz curve and the line of perfect equality quantifies the degree of inequality: smaller deviations from the line indicate a more equitable distribution^17^. In this study, the X-axis of the Lorenz curve represents the cumulative percentage of the population or geographic area, while the Y-axis corresponds to the cumulative percentage of PCCM departments.

### The Gini coefficient

As a quantitative extension of the Lorenz curve, the Gini coefficient quantifies the degree of PCCM departments' resource inequality by calculating the ratio of the area between the Lorenz curve and the line of perfect equality to the total area under the line of perfect equality. A widely used measure of income inequality, it ranges from 0 to 1 and is also commonly applied to evaluate the distribution of healthcare resources^18^. According to international standards, a Gini coefficient of 0 < Gini coefficient < 0.2 indicates perfect equality, 0.2 ≤ Gini coefficient < 0.3 represents relatively equal distribution, 0.3 ≤ Gini coefficient < 0.4 suggests moderate equality, 0.4 ≤ Gini coefficient < 0.5 denotes relative inequality, and 0.5 ≤ Gini coefficient < 1 signifies severe inequality. Here, “0” represents perfect equality, while “1” reflects maximum inequality. In this study, *n* represents the number of provinces (31 in total), *X*_i_ denotes the cumulative percentage of population or geographic area in the *i*-th province, and *Y*_i_ represents the cumulative percentage of PCCM departments in the *i*-th province.

$$G=1-{\sum }_{i=1}^{n}({X}_{i}+1-{X}_{i})({Y}_{i}+1+{Y}_{i})$$

In simple terms, this coefficient measures the deviation of PCCM departments' resource distribution from an ideally equitable state by calculating the ratio of the area between the Lorenz curve and the line of perfect equality to the total area under the line of perfect equality. For example, if the cumulative proportion of PCCM departments in a province exactly matches its population share, its contribution to the Gini coefficient is 0, representing absolute fairness.

### Theil index

The Theil index complements the Gini coefficient by decomposing overall inequality into inter-regional and intra-regional components, enabling identification of the primary sources of disparity—a key insight for guiding targeted policy interventions. First introduced by Dutch economist Theil in 1967, it is a widely used measure of income inequality across individuals or regions^19^. Over time, its application has expanded to analyze disparities in resource allocation among different regions. The Theil index is nonnegative, with larger values indicating greater inequality in resource distribution. In this study, the Theil index was used to evaluate population-based and geographic area-based inequality in PCCM department distribution and to decompose total inequality into inter-regional and intra-regional components. In this study, Pi represents the proportion of the population (or geographic area) in each province; *R* refers to the number of PCCM departments allocated by population (or area) across the 31 provinces; *R*_i_ denotes the total population (or geographic area) across all 31 provinces nationwide, and *n* is the number of provinces. The specific formula is as follows:

$$T={\sum }_{i=1}^{n}{P}_{i}\times \,\log \left(\frac{\bar{R}}{\bar{{R}_{i}}}\right)$$

To evaluate the differences within or between regions^20^, the Theil index formula can be transformed as follows:

$$T=T\,{{{\mathrm{int}}}}\,{er}+T\,{{{\mathrm{int}}}}\,{ra}$$

$$T\,{{{\mathrm{int}}}}\,{er}={\sum }_{j=1}^{m}{P}_{j}\times \,\log \left(\frac{{P}_{j}}{\bar{{Y}{j}}}\right)$$

$$T\,{{{\mathrm{int}}}}\,{ra}={\sum }_{j=1}^{m}{P}_{j}\times {T}_{j}$$

In this study, *P*_j_ represents the proportion of the population (or geographic area) of each of the six regions-North, Northeast, East, Central South, Southwest, and Northwest-relative to the total population (or geographic area) of the Chinese mainland. *Y*_j_ denotes the proportion of PCCM departments in each region relative to the total number of PCCM departments nationwide. *T*_j_ refers to the Theil index for each of the six regions, quantifying the degree of inequality in the distribution of PCCM departments within those regions.

The core of this index is to decompose overall inequality into inter-regional (e.g., between eastern and western regions of the Chinese mainland) and intra-regional (e.g., among provinces in the East region) differences, derived by summing the logarithmic product of each region’s population/area share and PCCM departments' resource share. For instance, if the PCCM departments share (27.7%) of the East region is close to its population share (29.9%), its contribution to overall inequality is relatively small. Because the Theil index contains a logarithmic term and requires careful treatment when resource shares are zero, temporal Theil comparisons were restricted to 2021–2023, when all provincial-level regions had nonzero PCCM department counts.

### PCCM Density Index

The Health Resource Density Index (HRDI) is a well-established metric for evaluating the distribution of health resources relative to both population size and geographic area. Building on the foundational principles of HRDI, this study introduces the PCCMDI to specifically assess the distribution of PCCM departments^21^. Derived by adapting the HRDI framework, the PCCMDI is calculated as the ratio of the number of PCCM departments in a given region to the geometric mean of that region’s population (in 10,000 persons) and geographic area (in 10,000 km²). This tailored adaptation ensures the PCCMDI effectively captures PCCM departments' accessibility by accounting for both population size and geographic scope. Mathematically, the index is defined as follows:

$${{{\mathrm{PCCMDI}}}}=\frac{PCCMi}{\sqrt{{A}_{i}\times {P}_{i}}}$$

This index integrates population size and geographic area to intuitively reflect the accessibility of PCCM departments' resources per unit population and unit area. For example, Shanghai, with a dense population and a small area, has a significantly higher PCCMDI (12.88) than Xizang (0.10), which is sparsely populated and geographically vast.

### Geographical detector

The geographical detector, developed by Wang et al., is a statistical method designed to identify spatial heterogeneity and uncover the underlying driving factors^22^. This method is widely used to analyze the influence mechanisms of social and environmental factors and comprises four types: risk detection, factor detection, ecological detection, and interaction detection^23^. In this study, at the provincial level in the Chinese mainland, factor detection and interaction detection were employed. In the geographical detector analysis, the study variable was the provincial distribution of PCCM departments in 2023, and the candidate factors were province-level demographic, economic, healthcare, educational, and administrative indicators. Factor detection evaluates whether spatial heterogeneity exists in the distribution of PCCM departments in the Chinese mainland and examines the extent to which the spatial stratification of each factor corresponds to this heterogeneity^24^. In the formula, *m* = 1, …, *L* represents the strata of factor *X* and variable *Y*; *N*_m_ and *N* denote the number of units in the *m*-th stratum and the entire area, respectively; *σ*²_*m* and *σ*² refer to the variance of *Y* values in the *m*-th stratum and the entire area, respectively. A larger *q*-value indicates stronger spatial correspondence between the stratification of factor *X* and the observed spatial variation in *Y*. The specific formula is as follows:

$$q=1-\frac{{\sum }_{m=1}^{L}{\sigma }_{m}^{2}{N}_{m}}{N{{{{\rm{\sigma }}}}}^{2}}$$

The *q*-value quantifies the degree of spatial stratified heterogeneity associated with a factor by comparing within-stratum variance with the overall variance of the study variable. For example, the *q*-value of resident population size (X1) is 0.815, indicating strong spatial correspondence between this factor and provincial variation in PCCM department distribution.

Interaction detection identifies the interaction between different factors, specifically examining whether the combined effect of two factors enhances, weakens, or independently influences their joint spatial correspondence with the study variable^25^. The results are categorized into five distinct types, as outlined in Supplementary Data 1 in the Online Data Supplement. In this framework, interaction refers to the enhancement or weakening of spatial stratified heterogeneity. Since geographical detector analysis typically requires candidate factors to be categorical, all factors were discretized and classified before analysis. The analysis was conducted using the GD package in R, with a *p*-value of <0.05 considered statistically significant.

### Statistics and reproducibility

All statistical analyses were performed using Microsoft Excel, R, and ArcGIS Pro 3.1. Descriptive statistics summarized the changes in the PCCM department distribution from 2018 to 2023. Inequality was assessed using Lorenz curves, Gini coefficients, Theil indices, and the PCCMDI, calculated at the provincial level (*n* = 31). Geodetector analysis was used to identify factors associated with spatial heterogeneity in provincial distribution; candidate factors were discretized into strata using the GD package in R, and *q*-values were calculated to quantify spatial correspondence. Statistical significance was set at *p* < 0.05, and all tests were two-sided where applicable. For comparisons across hospital types in Fig. 3D, E, two-sided Pearson’s *χ*² tests with Bonferroni correction were performed in R to adjust for multiple comparisons. No experimental replicates were applicable as this study analyzed population-level administrative data; data integrity was verified through cross-checking across three independent sources (China PCCM registry, health statistical yearbooks, and census databases).

## Results

### Expansion and regional trends in PCCM departments

**Fig. 2:**
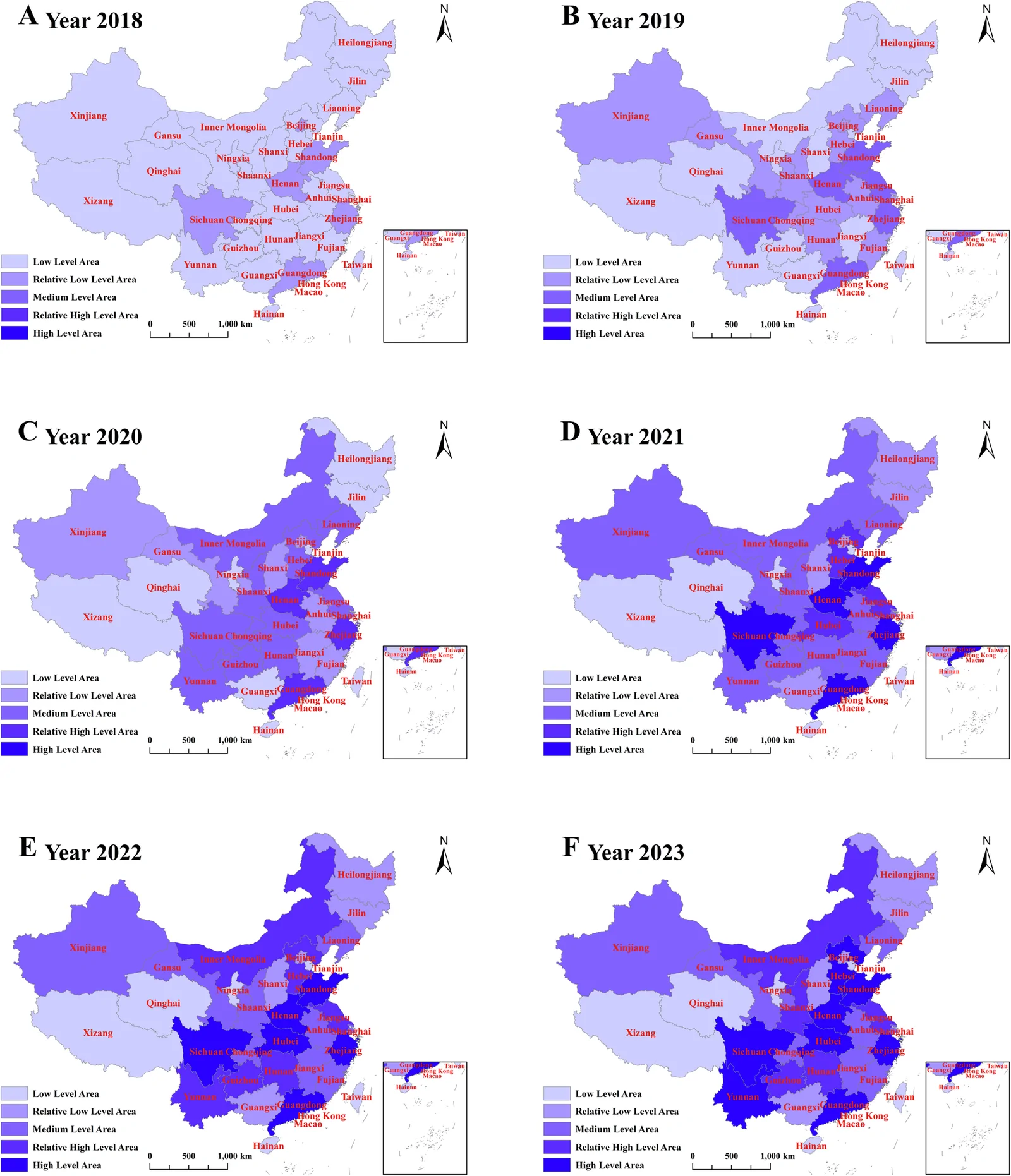
Temporal and spatial evolution of PCCM departments in the Chinese mainland (2018–2023). **A** Distribution of PCCM in the Chinese mainland in 2018. **B** Distribution of PCCM in the Chinese mainland in 2019. **C** Distribution of PCCM in the Chinese mainland in 2020. **D** Distribution of PCCM in the Chinese mainland in 2021. **E** Distribution of PCCM in the Chinese mainland in 2022. **F** Distribution of PCCM in the Chinese mainland in 2023. The base geographic boundary data for this map were obtained from the National Geographic Information Public Service Platform and released by the National Geomatics Center of China under map review approval no. GS(2024)0650.

The Chinese mainland has experienced a significant expansion in the number of PCCM departments over recent years. From 2018 to 2023, the number of PCCM departments increased from 409 to 2335, reflecting a growth rate (GR) of 470.90% and an average annual increase of 382.5. The Central South, Southwest, and Northwest regions demonstrated the most rapid growth, with GRs of 687.34%, 601.67%, and 505.71%, respectively (Table 1). Using the Jenks natural breaks classification method, the 31 provinces were grouped into five categories based on the number of PCCM departments: Low-level areas (0–19), Relative low-level areas (20–42), Medium-level areas (43–72), Relative high-level areas (73–104), and High-level areas (105–194). As shown in Fig. 2, the number of PCCM departments grew rapidly between 2018 and 2023. Eight provinces advanced to the High-level areas category, while five reached the Relative high level areas. The number of provinces classified as Medium level areas increased from none to eight, whereas the number of provinces in the Low level and Relative low level areas declined from 25 and 6 to 5 and 5, respectively.

**Table 1:** Quantity of PCCM departments in the Chinese mainland from 2018 to 2023

Region and province	2018	2019	2020	2021	2022	2023	GRs (%)
Nationwide	409	765	1325	1812	2121	2335	470.90
North	77	113	195	250	281	327	324.68
Beijing	25	34	37	38	38	41	64.00
Tianjin	10	16	18	18	18	18	80.00
Hebei	15	31	67	91	102	135	800.00
Shanxi	15	18	28	33	38	39	160.00
Inner Mongolia	12	14	45	70	85	94	683.33
Northeast	27	53	81	89	94	107	296.30
Liaoning	13	32	45	47	50	60	361.54
Jilin	8	10	19	20	20	20	150.00
Heilongjiang	6	11	17	22	24	27	350.00
East	131	238	397	550	616	646	393.13
Shanghai	17	33	47	50	51	51	200.00
Jiangsu	19	45	70	89	100	104	447.37
Zhejiang	34	48	87	120	127	131	285.29
Anhui	15	22	42	65	69	70	366.67
Fujian	16	24	30	46	66	72	350.00
Jiangxi	5	8	25	46	59	63	1160.00
Shandong	25	58	96	134	144	155	520.00
Central South	79	163	308	444	579	622	687.34
Henan	28	51	102	133	154	158	464.29
Hubei	10	23	54	92	127	137	1270.00
Hunan	9	30	48	68	90	100	1011.11
Guangdong	29	49	85	126	181	194	568.97
Guangxi	3	8	17	23	25	30	900.00
Hainan	0	2	2	2	2	3	50.00
Southwest	60	103	208	297	353	421	601.67
Chongqing	9	23	43	52	58	65	622.22
Sichuan	32	49	60	105	129	156	387.50
Guizhou	9	16	51	70	78	80	788.89
Yunnan	10	15	54	69	86	118	1080.00
Xizang	0	0	0	1	2	2	100.00
Northwest	35	94	136	182	198	212	505.71
Shaanxi	15	36	50	60	66	73	386.67
Gansu	3	20	33	49	54	59	1866.67
Qinghai	1	2	7	7	7	8	700.00
Ningxia	2	9	14	16	17	17	750.00
Xinjiang	14	28	32	50	54	55	292.86

*GRs* the growth rates.

### Provincial and regional distribution of PCCM departments in the Chinese mainland

As of 2023, a total of 2335 hospitals in the Chinese mainland have established PCCM departments, spanning all 31 provincial-level regions. At the provincial level, the top five provinces with the highest number of PCCM are Guangdong (194), Henan (158), Sichuan (156), Shandong (155), and Hubei (137). Regionally, the distribution of PCCM is as follows: East (646), Central South (622), Southwest (421), North (327), Northwest (212), and Northeast (107) (Table 2). The national average number of PCCM departments per million population in 2023 is 1.66. Provincially, the top five provinces in PCCM per million population are Inner Mongolia (3.92), Yunnan (2.53), Gansu (2.39), Hubei (2.35), and Ningxia (2.33). Regionally, the rankings are: Southwest (2.06), Northwest (2.05), North (1.95), Central South (1.52), East (1.52), and Northeast (1.12). In terms of PCCM departments per 10,000 square kilometers, the top five provinces and municipalities are Shanghai (80.95), Beijing (25.00), Tianjin (15.00), Zhejiang (12.42), and Guangdong (10.79). Regionally, the rankings are: East (7.99), Central South (6.11), North (2.10), Southwest (1.80), Northeast (1.32), and Northwest (0.69). The national PCCMDI in 2023 is 2.01, with 21 provinces exceeding this average. The top five provinces by PCCMDI are Shanghai (12.88), Beijing (6.85), Zhejiang (4.95), Tianjin (4.45), and Hubei (4.16) (Table 2).

**Table 2:** Distribution of PCCM departments in the Chinese mainland in 2023

Region and province	Population (10,000 persons)	Total area (10,000 km^2^)	PCCM departments	PCCM departments		PCCMDI
				Per million persons	Per 10,000 km^2^	
Nationwide	140,767	961.81	2335	1.66	2.43	2.01
North	16,805	155.69	327	1.95	2.10	2.02
Beijing	2186	1.64	41	1.88	25.00	6.85
Tianjin	1364	1.2	18	1.32	15.00	4.45
Hebei	7393	18.88	135	1.83	7.15	3.61
Shanxi	3466	15.67	39	1.13	2.49	1.67
Inner Mongolia	2396	118.3	94	3.92	0.79	1.77
Northeast	9583	80.91	107	1.12	1.32	1.22
Liaoning	4182	14.87	60	1.43	4.03	2.41
Jilin	2339	18.74	20	0.86	1.07	0.96
Heilongjiang	3062	47.3	27	0.88	0.57	0.71
East	42,582	80.81	646	1.52	7.99	3.48
Shanghai	2487	0.63	51	2.05	80.95	12.88
Jiangsu	8526	10.72	104	1.22	9.70	3.44
Zhejiang	6627	10.55	131	1.98	12.42	4.95
Anhui	6121	14.01	70	1.14	5.00	2.39
Fujian	4183	12.4	72	1.72	5.81	3.16
Jiangxi	4515	16.69	63	1.40	3.77	2.30
Shandong	10,123	15.81	155	1.53	9.80	3.87
Central South	40,997	101.75	622	1.52	6.11	3.05
Henan	9815	16.7	158	1.61	9.46	3.90
Hubei	5838	18.59	137	2.35	7.37	4.16
Hunan	6568	21.18	100	1.52	4.72	2.68
Guangdong	12,706	17.98	194	1.53	10.79	4.06
Guangxi	5027	23.76	30	0.60	1.26	0.87
Hainan	1043	3.54	3	0.29	0.85	0.49
Southwest	20,462	234.15	421	2.06	1.80	1.92
Chongqing	3191	8.24	65	2.04	7.89	4.01
Sichuan	8368	48.6	156	1.86	3.21	2.45
Guizhou	3865	17.62	80	2.07	4.54	3.07
Yunnan	4673	39.41	118	2.53	2.99	2.75
Xizang	365	120.28	2	0.55	0.02	0.10
Northwest	10,338	308.5	212	2.05	0.69	1.19
Shaanxi	3952	20.56	73	1.85	3.55	2.56
Gansu	2465	42.58	59	2.39	1.39	1.82
Qinghai	594	72.23	8	1.35	0.11	0.39
Ningxia	729	6.64	17	2.33	2.56	2.44
Xinjiang	2598	166.49	55	2.12	0.33	0.84

*PCCMDI* the PCCM Department Density Index.

Data from 2335 hospitals show that PCCM departments consist of an average of 78.29 beds (including RICU beds), 14.30 doctors, and 26.78 nurses. Upon further investigation, the nurse-to-physician ratio is found to be 1.96, while the physician-to-bed ratio stands at 0.18. Additionally, the nurse-to-bed ratio is 0.36. Remarkably, all these indicators fall below the standard thresholds (Table 3).

**Table 3:** Description of PCCM doctors, nurses, and beds across different regions and provinces in the Chinese mainland

Region and province	Average number of beds	Doctors	Nurses	Physician-to-bed ratio	Nurse-to-bed ratio	Nurse-to-physician ratio
Nationwide	78.29	14.30	26.78	0.18	0.36	1.96
North	72.10	14.84	27.27	0.21	0.41	1.96
Beijing	79.04	19.86	42.79	0.26	0.55	2.11
Tianjin	78.92	26.67	31.32	0.34	0.39	1.18
Hebei	67.36	14.37	24.44	0.19	0.35	1.82
Shanxi	85.19	15.53	27.94	0.21	0.38	1.78
Inner Mongolia	63.62	10.44	21.84	0.19	0.41	2.17
Northeast	77.14	12.73	21.26	0.17	0.32	1.86
Liaoning	78.40	12.52	19.83	0.14	0.28	2.05
Jilin	49.82	10.40	17.50	0.20	0.32	1.63
Heilongjiang	100.45	16.25	28.25	0.22	0.38	1.75
East	82.56	16.30	29.08	0.20	0.37	1.82
Shanghai	64.89	18.56	24.88	0.26	0.41	1.60
Jiangsu	87.05	17.47	31.12	0.23	0.41	1.82
Zhejiang	75.39	15.88	28.29	0.20	0.37	1.81
Anhui	81.49	16.65	29.73	0.20	0.33	1.67
Fujian	85.17	14.93	28.05	0.19	0.37	1.93
Jiangxi	99.34	14.97	35.45	0.16	0.36	2.19
Shandong	83.23	16.17	27.10	0.21	0.37	1.77
Central South	77.06	13.44	28.35	0.18	0.39	2.12
Henan	74.44	11.86	24.57	0.17	0.36	2.07
Hubei	82.84	13.52	25.38	0.19	0.37	1.97
Hunan	89.31	14.83	32.28	0.18	0.38	2.17
Guangdong	66.58	13.03	26.78	0.18	0.38	2.08
Guangxi	79.08	16.10	41.56	0.20	0.49	2.49
Hainan	96.00	23.00	72.00	0.24	0.75	3.13
Southwest	82.78	13.39	24.84	0.16	0.31	1.98
Chongqing	91.47	14.57	26.38	0.16	0.31	1.94
Sichuan	96.85	14.83	28.23	0.15	0.31	2.10
Guizhou	71.49	13.22	22.79	0.17	0.31	1.83
Yunnan	65.85	10.66	20.85	0.16	0.32	1.92
Xizang	35.00	9.00	11.50	0.26	0.33	1.28
Northwest	67.27	11.97	22.90	0.18	0.35	1.94
Shaanxi	69.52	11.93	23.80	0.19	0.35	1.84
Gansu	61.79	10.18	20.32	0.17	0.34	1.99
Qinghai	71.17	12.40	27.75	0.16	0.35	2.18
Ningxia	59.42	12.44	19.14	0.24	0.36	1.52
Xinjiang	74.90	14.25	25.42	0.19	0.32	1.72

### Equity based on the Lorenz curve and the Gini coefficient

**Fig. 3:**
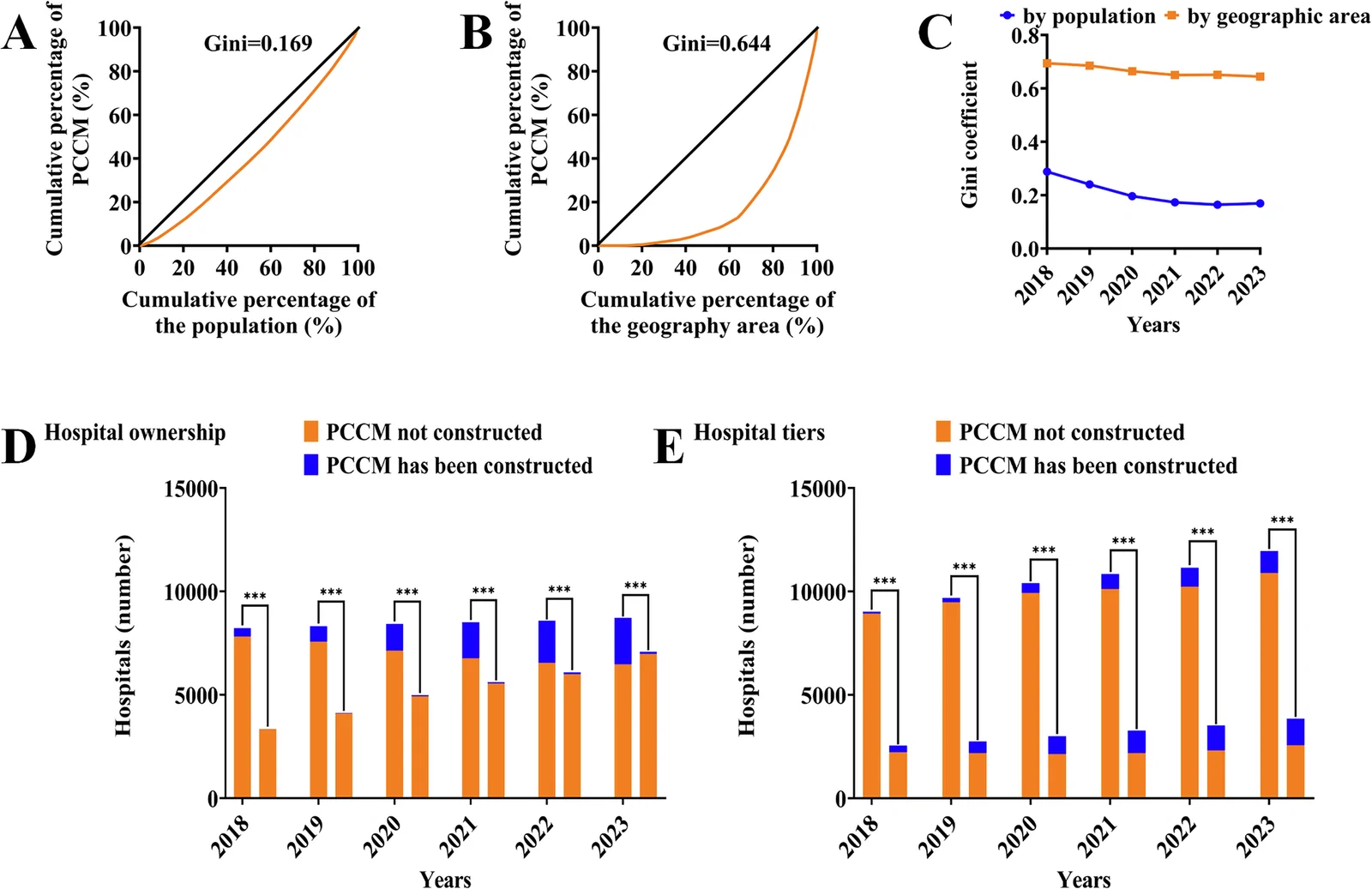
Equity analysis of PCCM distribution in the Chinese mainland: population-based and geographic disparities (2018–2023). **A** Lorenz curve illustrating the near-equitable distribution of PCCM departments relative to population. **B** Lorenz curve demonstrating significant geographic inequities in PCCM distribution. **C** Temporal trends in Gini coefficients for population-based and geographic distribution of PCCM, highlighting persistent geographic disparities. **D** Temporal distribution of PCCM construction across hospital ownership types from 2018 to 2023 (public vs. private), assessed using two-sided Pearson’s *χ*² tests with Bonferroni correction; adjusted *P* values were 1.69 × 10^−^^35^, 3.52 × 10^−^^73^, 5.04 × 10^−^^158^, 3.10 ×  10^−^^242^, 8.66 × 10^−^^312^, and <1 × 10^−^^323^, respectively. **E** Temporal distribution of PCCM construction across hospital tiers from 2018 to 2023 (secondary vs tertiary), assessed using two-sided Pearson’s *χ*² tests with Bonferroni correction; adjusted *P* values were 8.00 × 10^−^^171^, 1.59 × 10^−^^264^, <1 × 10^−^^323^, <1 × 10^−^^323^, <1 × 10^−^^323^, and 2.43 × 10^−^^304^, respectively.

The Lorenz curve and Gini coefficient are inherently complementary tools for assessing distribution equity: the Lorenz curve graphically depicts the cumulative proportion of PCCM departments' resources against the cumulative proportion of population or geographic area, while the Gini coefficient quantifies the degree of inequality by measuring the area between the Lorenz curve and the line of perfect equality (a smaller value indicates higher equity). First, regarding population-based equity in 2023 (Fig. 3A), the Lorenz curve closely aligns with the line of perfect equality, corresponding to a Gini coefficient of 0.169, which falls within the range of near-perfect equality and indicates that PCCM departments' distribution is highly balanced relative to population size. In contrast, the geographic-based Lorenz curve (Fig. 3B) deviates significantly from the line of perfect equality, with a Gini coefficient of 0.644, reflecting severe inequity in the spatial accessibility of PCCM departments' resources.

To evaluate temporal trends in equity, Gini coefficients were compared between 2018 and 2023 (Fig. 3C). The population-based Gini coefficient decreased from 0.288 to 0.169, shifting from relatively equal to near-perfect equality and demonstrating substantial progress in narrowing population-related disparities. However, the geographic-based Gini coefficient only slightly declined from 0.694 to 0.644, remaining in the severe inequality range, indicating persistent structural imbalances in geographic resource allocation.

According to the functions, facilities, and technical capabilities of hospitals in the Chinese mainland, they are categorized into first-level hospitals, second-level hospitals, and third-level hospitals. From 2018 to 2023, the proportion of hospitals with PCCM departments increased across both hospital tiers and ownership types, but remained consistently higher in tertiary than in secondary hospitals and in public than in private hospitals (Fig. 3D, E). PCCM coverage increased from 0.99 to 8.79% in secondary hospitals and from 12.56 to 33.33% in tertiary hospitals; over the same period, it increased from 4.91 to 25.68% in public hospitals and from 0.15 to 1.36% in private hospitals. All between-group differences were significant in each year (two-sided Pearson’s *χ*² tests with Bonferroni correction; all adjusted *P* < 0.001). Beyond hospital-level tiering, the PCCM accreditation system implemented in the Chinese mainland further categorizes PCCM departments into four hierarchical levels based on infrastructure, technical capacity, and service quality: cultivation units (30), qualified units (1459), excellent units (817), and demonstration units (29) (Table 4). This dual-tiered framework (hospital level and department level) constitutes the core institutional design for balancing national quality standards with regional adaptability, which is central to the study’s analysis of population-based equity.

**Table 4:** Characteristics of the PCCM departments in the Chinese mainland in 2023

Region and province	Cultivation unit	Qualified units	Excellent unit	Demonstration unit
Nationwide	30	1459	817	29
North	8	210	104	5
Beijing	0	24	12	5
Tianjin	0	12	6	0
Hebei	1	67	67	0
Shanxi	2	30	7	0
Inner Mongolia	5	77	12	0
Northeast	3	85	18	1
Liaoning	1	48	10	1
Jilin	0	14	6	0
Heilongjiang	2	23	2	0
East	4	354	274	14
Shanghai	0	15	29	7
Jiangsu	0	60	43	1
Zhejiang	2	82	47	0
Anhui	0	46	23	1
Fujian	0	36	35	1
Jiangxi	1	45	17	0
Shandong	1	70	80	4
Central South	5	365	246	6
Henan	0	74	84	0
Hubei	1	74	61	1
Hunan	0	66	33	1
Guangdong	4	126	60	4
Guangxi	0	23	7	0
Hainan	0	2	1	0
Southwest	8	286	124	3
Chongqing	0	30	33	2
Sichuan	5	126	24	1
Guizhou	1	50	29	0
Yunnan	2	78	38	0
Xizang	0	2	0	0
Northwest	2	159	51	0
Shaanxi	0	49	24	0
Gansu	1	44	14	0
Qinghai	0	8	0	0
Ningxia	1	11	5	0
Xinjiang	0	47	8	0

### Regional inequality in the geographic distribution of PCCM departments in the Chinese mainland

**Fig. 4:**
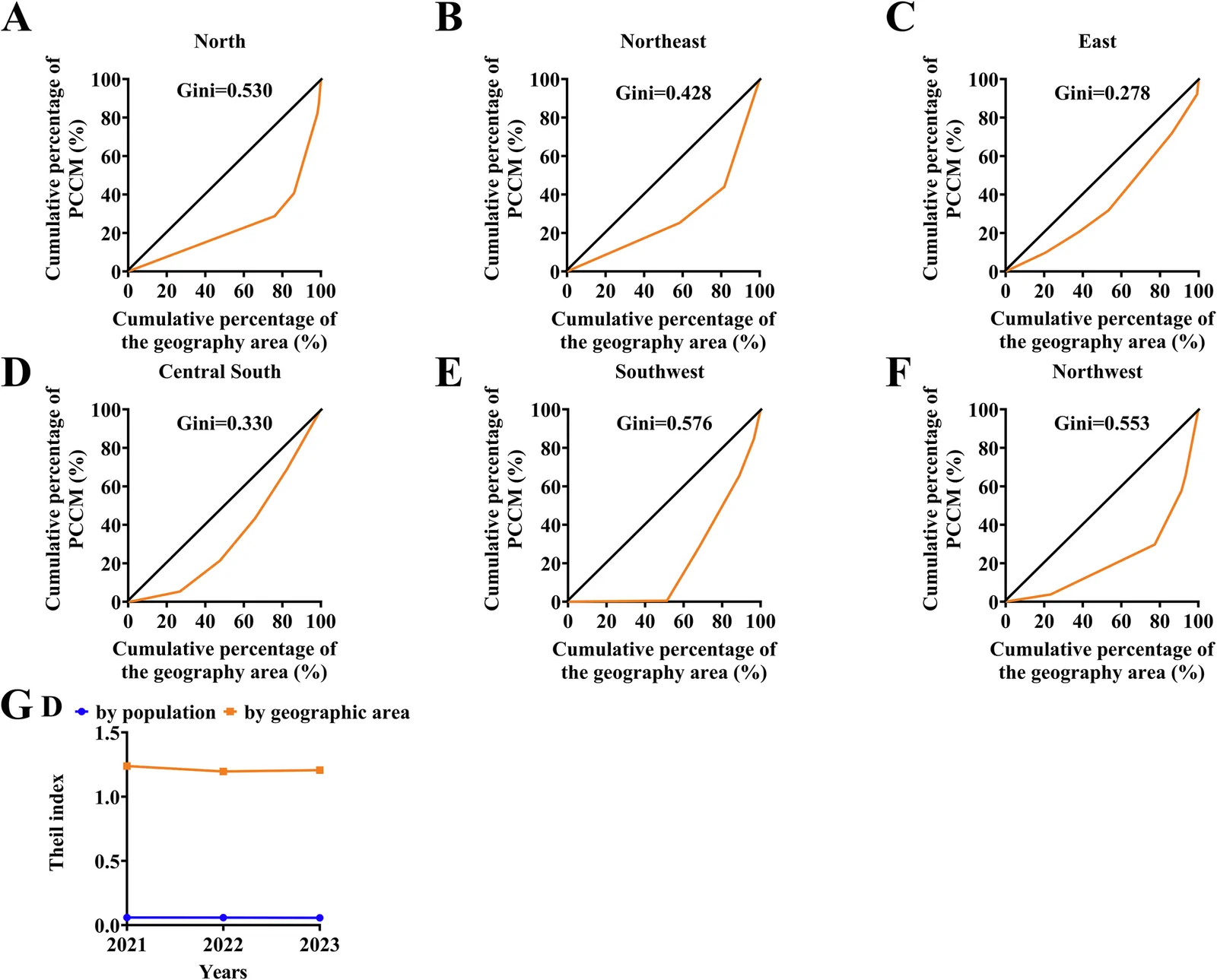
Lorenz curves and Gini coefficients of PCCM by geographic area in different regions in 2023. **A** Lorenz curve and Gini coefficients of PCCM by geographic area in North in 2023. **B** Lorenz curves and Gini coefficients of PCCM by geographic area in the Northeast in 2023. **C** Lorenz curves and Gini coefficients of PCCM by geographic area in the East in 2023. **D** Lorenz curves and Gini coefficients of PCCM by geographic area in Central South in 2023. **E** Lorenz curves and Gini coefficients of PCCM by geographic area in the Southwest in 2023. **F** Lorenz curves and Gini coefficients of PCCM by geographic area in the Northwest in 2023. **G** Inequality trends based on the Theil index in the Chinese mainland from 2021 to 2023.

To further analyze disparities in the geographic distribution of PCCM departments, the six major regions of the Chinese mainland were examined. The findings revealed that the East region exhibited relative equity (Gini = 0.278), Central South demonstrated moderate equity (Gini = 0.330), and the Northeast displayed relative inequity (Gini = 0.428). In contrast, the North, Southwest, and Northwest regions showed severe inequity, with Gini indices of 0.530, 0.576, and 0.553, respectively. These results indicate that the primary sources of geographic inequity in the distribution of PCCM departments are concentrated in the North, Southwest, and Northwest regions (Fig. 4A–F).

**Fig. 5:**
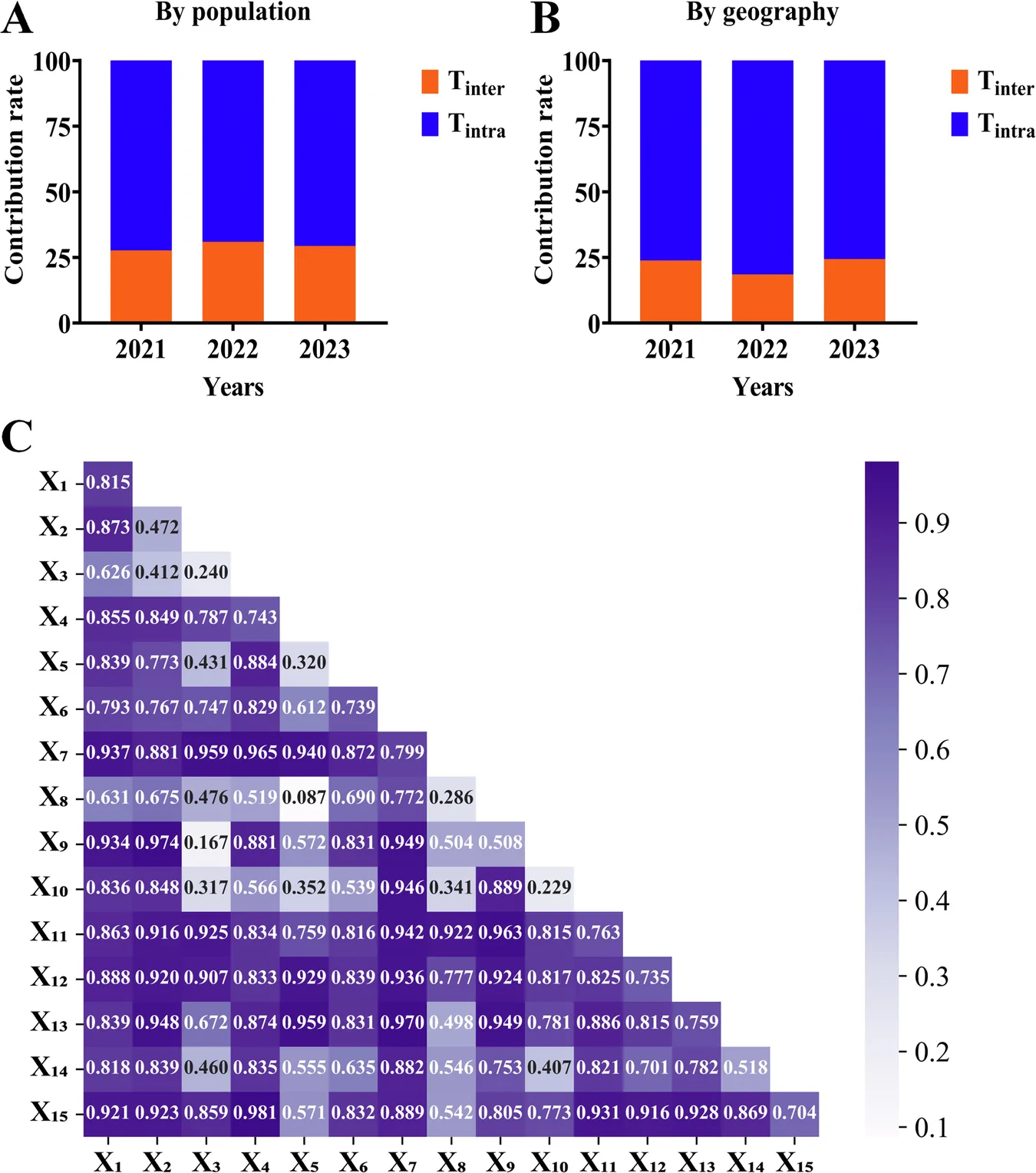
Decomposition of regional inequality and interaction patterns in PCCM distribution across the Chinese mainland. **A** Contributions of inter-regional and intra-regional disparities to population-based inequality in PCCM distribution. **B** Contributions of inter-regional and intra-regional disparities to geographic area-based inequality in PCCM distribution. **C** Interaction patterns of key determinants (e.g., GDP, public hospital density, and government health expenditure) on the spatial allocation of PCCM departments, highlighting synergistic influences on regional disparities.

In 2023, the Theil index for PCCM departments based on population distribution was 0.057, and that based on geographical area distribution was 1.206. To evaluate recent temporal patterns using a consistent calculation framework, we compared Theil indices from 2021 to 2023: the population-based index decreased from 0.059 to 0.057, while the geographical area-based index declined slightly from 1.238 to 1.206 (Fig. 4G). Decomposition of the Theil index further characterized the relative contributions of within-region and between-region components under the six-region classification used in this study (Fig. 5A, B). Together with the regional geographic Gini coefficients, these findings indicate that residual inequality in PCCM department distribution remained evident, particularly when geographic area was considered.

### Factors associated with spatial heterogeneity in PCCM department distribution

In 2023, the distribution of PCCM departments in the Chinese mainland demonstrated significant spatial heterogeneity. Among the 15 evaluated factors, the five factors showing the strongest spatial correspondence with PCCM departments distribution (ranked by *q*-value) were: (1) size of resident population (*q* = 0.815), (2) number of public hospitals (*q* = 0.799), (3) government health expenditure (*q* = 0.763), (4) proportion of the population with a college degree or above (*q* = 0.759), and (5) GDP (*q* = 0.743), with all results being statistically significant (*p* < 0.0001). These variables collectively were strongly associated with spatial disparities in PCCM department distribution.

While individual factors exerted significant influences, the distribution of PCCM departments is better understood as the outcome of complex spatial interplay between multiple factors. Interaction detection revealed that most pairwise interactions were categorized as “enhance, bivariate” or “enhance, nonlinear,” indicating that the joint spatial correspondence of two factors exceeded that of individual factors. The top five interaction patterns were: GDP and number of prefecture-level cities (*q* = 0.981), population density and medical insurance depth (*q* = 0.974), number of public hospitals and proportion of the population with a college degree or above (*q* = 0.970), GDP and number of public hospitals (*q* = 0.965), and medical insurance depth and government health expenditure (*q* = 0.963). Notably, even factors with limited individual *q*-values (e.g., population density, medical insurance depth) exhibited substantial enhancement in joint spatial correspondence when combined, highlighting the multi-factorial nature of PCCM departments' distribution.

## Discussion

The rapid scaling of PCCM in the Chinese mainland from 2018 to 2023 offers critical insights into how institutional innovations can address the dual challenges of healthcare equity and scalability in a vast, heterogeneous setting. Central to this success is the tiered accreditation system, a policy mechanism that diverges from traditional top-down standardization by allowing hospitals to meet phased benchmarks aligned with local capacities. This approach not only spurred a 470.9% growth in PCCM departments but also achieved near-equitable population-based distribution (Gini: 0.169), a feat rarely observed in low- and middle-income countries (LMICs) grappling with similar disparities. Below, we contextualize these findings within global health policy debates, analyze the drivers and limitations of the model implemented in the Chinese mainland, and propose actionable lessons for respiratory care systems worldwide.

Institutional flexibility as a catalyst for equity. The tiered accreditation framework—categorizing hospitals into secondary and tertiary tiers with differentiated requirements—proved instrumental in balancing national quality standards with regional realities (Supplementary Data 2). Rather than enforcing a one-size-fits-all benchmark for PCCM practice nationwide, our tiered accreditation system stratifies departments into four progressive levels—cultivation, qualified, excellent, and demonstration units—with tailored criteria calibrated to local institutional capacity and regional healthcare needs; this gradient design empowers high-performing excellent and demonstration units to act as regional clinical and operational benchmarks, providing technical guidance and capacity-building support for lower-tier units, while also establishing a clear upward development pathway that incentivizes basic-level PCCM departments to continuously advance their infrastructure, technical capabilities and service quality toward higher accreditation standards. For instance, while tertiary hospitals in urban hubs like Beijing and Shanghai focused on advanced critical care integration, secondary hospitals in provinces like Yunnan prioritized basic PCCM departments' infrastructure. This flexibility enabled underserved regions to achieve GRs exceeding 600% (e.g., the Southwest region), narrowing population-based gaps without overwhelming resource-limited settings. Crucially, our geographical detector analysis showed that provincial variation in PCCM departments' distribution was strongly patterned by population size, public hospital availability, and government health expenditure, underscoring that localized capacity and institutional context, rather than a uniform implementation pathway alone, were closely associated with equity gains. This contrasts sharply with the U.S. model, where rigid specialization standards often exacerbate urban-rural divides. The experience of the Chinese mainland suggests that decentralized implementation, guided by national societies like the CACP, can mitigate such imbalances. By delegating accreditation oversight to provincial authorities while maintaining central quality audits, the governance model in the Chinese mainland harmonized scalability with accountability—a strategy absent in many LMICs struggling with fragmented governance.

Synergies between economic and health policies. The integration of health infrastructure development with broader economic strategies emerged as another cornerstone of success^26^. Interaction patterns between GDP and administrative divisions (*q* = 0.981) revealed that provinces with robust economic-health policy alignment (e.g., Guangdong, Shandong) optimized PCCM departments' resource allocation, whereas regions relying solely on health-sector interventions lagged. For example, Inner Mongolia’s high per capita PCCM density (3.92/million) stemmed from targeted investments in rural healthcare under its regional poverty alleviation agenda, not merely hospital-level initiatives. However, the persistence of severe geographic disparities (Gini: 0.644) signals the limits of economic-driven approaches. Wealthier provinces dominated absolute PCCM department numbers, while remote areas, such as Xizang, remained critically underserved despite high per capita metrics-a paradox highlighting that population-based equity does not guarantee geographic accessibility. This mirrors challenges observed in Brazil’s SUS system and India’s NHM, where infrastructure clustering in urban centers perpetuates rural neglect^27,28^.

Workforce shortages and care quality deficits. While the model implemented in the Chinese mainland excelled in infrastructure expansion, workforce shortages-evidenced by suboptimal nurse-to-physician (1.96) and physician-to-bed (0.18) ratios-reveal systemic gaps in human capital investment^29^. These ratios fall markedly below WHO recommendations and U.S. benchmarks (e.g., nurse-to-physician ratio: 3.1), impairing care quality and sustainability. Similar issues plague LMICs like South Africa and Nigeria^30,31^, where PCCM departments' growth often outpaces staff training. Recent initiatives in the Chinese mainland to incentivize rural medical employment and expand telemedicine networks may offer remedies, but their long-term efficacy requires rigorous evaluation^32,33^.

The integration of economic policies with health infrastructure planning was central to addressing geographic disparities in PCCM department expansion across the Chinese mainland. Provinces, such as Guangdong and Shandong, exemplify successful integration, where regional economic development strategies-including the Greater Bay Area initiative and “strong counties” decentralization-explicitly incorporated health infrastructure nodes, resulting in optimized PCCM department distribution along economic corridors and improved geographic coverage. Conversely, Xizang illustrates the limits of purely fiscal integration: despite substantial central transfers, extreme terrain and low population density perpetuate accessibility gaps (PCCMDI 0.10), underscoring that economic resources must be paired with tailored delivery models^34^. The rapid expansion of PCCM departments alongside persistent workforce shortages reflects a deliberate phased strategy prioritizing infrastructure establishment, with human capital development now addressed through targeted interventions including the “Three-Tier” training framework (specialized training, advanced studies, single-skill modules), rural incentive programs (salary supplements, priority admission for underserved candidates), and telemedicine-supported distributed workforce models^35^. While our cross-sectional design identifies associations rather than definitive causal relationships, temporal precedence (post-2018 expansion), heterogeneous growth patterns consistent with staggered implementation, and geographical detector interaction patterns (e.g., GDP × administrative divisions, *q* = 0.981) collectively support the plausibility of tiered accreditation’s contribution. These findings should be interpreted as evidence of spatial patterning rather than as estimates of independent net effects. For other countries seeking to adapt the model implemented in the Chinese mainland, essential enabling factors include governance structures supporting central-local coordination, earmarked financing mechanisms, functional data infrastructure, and mature professional societies. In decentralized systems, central governments may shift from mandating to incentivizing adoption through conditional transfers; in privatized systems, accreditation can serve as a market signal influencing patient choice and insurance contracting. Ultimately, the experience of the Chinese mainland offers transferable principles-tiered progression, data-driven targeting, fiscal alignment, and hybrid governance—that require contextual adaptation rather than wholesale replication^36^.

This study has several limitations. First, reliance on provincial-level data may obscure intra-regional disparities, particularly in rural versus urban settings. Second, while geographical detector analysis identifies factors associated with spatial heterogeneity, causal relationships remain inferential due to the cross-sectional design. Third, the focus on quantitative distribution overlooks qualitative dimensions, such as care quality, patient outcomes, or interdisciplinary integration factors critical to equitable access. Future research should employ mixed-methods approaches to evaluate how distributional equity translates into clinical effectiveness, particularly in marginalized populations. Fourth, factors such as age demographics, disease burden, and healthcare-seeking behavior that may affect PCCM distribution were not included in the analysis due to our focus on macro-level institutional and socioeconomic determinants. In addition, standardized hospital-level service utilization data were not available; therefore, this study could not assess changes in outpatient volume or formally evaluate supply-demand matching for PCCM services.

## Conclusion

The experience of the Chinese mainland demonstrates that institutional flexibility—manifested through decentralized implementation and adaptive standards—can reconcile large-scale expansion with equity goals, offering a reform blueprint for the global community, particularly resource-constrained nations, to align national frameworks with localized realities.

## Supplementary information

Description of Additional Supplementary Files

Supplementary Data 1

Supplementary Data 2

Supplementary Data 3

Supplementary Data 4

Supplementary Data 5

Supplementary Data 6

## Data Availability

The datasets analyzed in this study are derived from publicly accessible sources. The China PCCM registry data are accessible at https://pccm.hxylt.org.cn/f (no registration required). The China Health Statistics Yearbooks are available from the National Health Commission at http://www.nhc.gov.cn/mohwsbwstjxxzx/tjzxtjsj/tjsj_list.shtml. The China Statistical Yearbooks are available from the National Bureau of Statistics at https://www.stats.gov.cn/sj/ndsj/. Hospital-level information on physicians, nurses, and beds was manually extracted from publicly accessible hospital websites because no centralized official dataset was available for these variables. The hospital website sources used for this extraction are provided in Supplementary Data 2. The curated hospital-level extraction dataset supporting the aggregate results in Table 3 is not deposited in a public repository because it was compiled from heterogeneous third-party hospital websites, and the authors are not the authoritative source for redistribution of these hospital-published data as a consolidated public database. The curated extraction dataset is available from the corresponding author (Dr. Weixin Chen, weixin011110@126.com) upon reasonable request for verification and academic reuse. No datasets with DOIs or accession codes were generated or used. Source data underlying all main and Supplementary Figs. are provided in Supplementary Data 3.

## Code availability

The Excel workbook containing the formula-based calculations and Geodetector input data is provided in Supplementary Data 4. The R code used for the two-sided Pearson’s *χ*² tests with Bonferroni correction and the Geodetector analysis is provided in Supplementary Data 5 and 6, respectively. ArcGIS project files are not redistributed because the base map data are subject to redistribution restrictions; the provincial-level PCCM department data used for map generation are provided in the Supplementary files, and the base map data can be independently obtained from the National Geographic Information Public Service Platform.
