# Supplementary material for: Using tiered accreditation and decentralized governance to achieve population-based equity in pulmonary and critical care medicine in the Chinese mainland
Source: Commun Med (Lond). 2026 Jun 10;6:496. doi: 10.1038/s43856-026-01724-1 (PMC13601544; doi:10.1038/s43856-026-01724-1)
Supplement: Supplementary file 1 [file 43856_2026_1724_MOESM1_ESM.pdf]

Supplementary Information

Description of Supplementary Files

The supplementary files provide detailed supporting information for this study, including definitions of variables used in the analysis, standardized evaluation criteria and data sources, and the source data underlying the main figures.

Supplementary Data 1. Definitions of explanatory variables and interaction categories used in the Geodetector analysis.

This file contains the detailed definitions, units, and factor codes for all explanatory variables included in the study, covering demographic, economic, hospital, medical insurance, health expenditure, educational, and administrative factors. It also provides the classification framework for the five types of interaction effects used to interpret Geodetector interaction detection results.

Supplementary Data 2. PCCM standardized construction criteria and hospital website sources.

This file includes the standardized construction and assessment criteria for PCCM departments in tertiary hospitals and secondary hospitals, forming the institutional basis for the tiered accreditation framework analyzed in this study. In addition, it provides the official website links of hospitals with established PCCM departments that were used to collect department-level information on physicians, nurses, and beds.

Supplementary Data 3. Source data underlying the main figures.

This file provides the source data for Figures 1–5 in the main text, including provincial classification by region, annual numbers of PCCM departments from 2018 to 2023, Lorenz curve data, Gini coefficients, Theil indices, comparisons by hospital tier and ownership, decomposition of inter- and intra-regional inequality, and interaction effects of key determinants on the spatial distribution of PCCM departments.

Supplementary Data 4. Excel workbook for formula-based equity analyses, PCCM density calculations, and Geodetector input data.

This file contains the Excel calculation sheets, formulas, intermediate values, and final results for the formula-based analyses, including Lorenz curves, Gini coefficients, Theil indices, and PCCM Density Index calculations. It also includes the provincial-level input dataset used for the Geodetector analysis, including PCCM department distribution data and corresponding explanatory variables.

Supplementary Data 5. R code for two-sided Pearson's chi-square tests with Bonferroni correction.

This file provides the R code used to perform two-sided Pearson's chi-square tests and Bonferroni adjustment for multiple comparisons. The code was used to assess between -group differences in PCCM department construction across hospital categories, as reported in the main text.

Supplementary Data 6. R code for Geodetector analysis of spatial heterogeneity in PCCM department distribution.

This file provides the R code used to conduct the Geodetector analysis. The code imports the provincial-level dataset, applies multiple discretization methods and interval settings to the continuous explanatory variables, and estimates the spatial correspondence between candidate factors and the provincial distribution of PCCM departments.
